# Effects of upper-extremity vascular access creation on cardiac events in patients undergoing coronary artery bypass grafting

**DOI:** 10.1371/journal.pone.0184168

**Published:** 2017-09-05

**Authors:** Youngjin Han, Suk Jung Choo, Hyunwook Kwon, Jae Won Lee, Cheol Hyun Chung, Hyangkyoung Kim, Tae-Won Kwon, Yong-Pil Cho

**Affiliations:** 1 Department of and Surgery, University of Ulsan College of Medicine and Asan Medical Center, Seoul, South Korea; 2 Department of Thoracic Surgery, University of Ulsan College of Medicine and Asan Medical Center, Seoul, South Korea; 3 Department of Surgery, Chung-Ang University Hospital, Seoul, South Korea; Universidade de Mogi das Cruzes, BRAZIL

## Abstract

The present study was conducted to investigate whether upper-extremity vascular access (VA) creation increases the risk for major adverse cardiac events (MACE) and death in patients undergoing coronary artery bypass grafting (CABG) with an *in situ* left internal thoracic artery (ITA) graft. A total of 111 patients with CABG with a left ITA graft who underwent upper-extremity VA creation were analyzed retrospectively; 93 patients received left VA creation (83.8%, ipsilateral group) and 18 patients received right VA creation (16.2%, contralateral group). The primary outcome was the occurrence of MACE, and the secondary outcome was the composite of MACE or late death. There were no significant differences in the incidence of primary (P = 0.30) or secondary (P = 0.09) outcomes between the two groups. Multivariate regression analysis indicated that prior cerebrovascular accidents (hazard ratio [HR] 3.30; 95% confidence interval [CI] 1.37–7.97; P = 0.01) and type of VA (HR 3.44; 95% CI 1.34–8.82; P = 0.01) were independently associated with MACE; prior peripheral arterial occlusive disease (HR 4.22; 95% CI 1.62–10.98; P<0.01) and type of VA (arteriovenous fistula *vs*. prosthetic arteriovenous grafting) (HR 3.06; 95% CI, 1.42–6.61; P<0.01) were associated with the composite of MACE or death. The side and location of VA were not associated with MACE or death. Our study showed no definite evidence that ipsilateral VA creation affects the subsequent occurrence of MACE or late death from any cause. The type of VA (a prosthetic arteriovenous grafting) is a significant predictor of the subsequent occurrence of MACE or late death.

## Introduction

Both chronic kidney disease (CKD) and symptomatic coronary artery disease (CAD) are associated with cardiovascular risk factors. The increase in the population with CKD and the extended life expectancy of patients with symptomatic CAD undergoing coronary artery bypass grafting (CABG) have resulted in more patients with CKD receiving CABG, especially those with diabetes mellitus and advanced age, being referred for vascular access (VA) creation for hemodialysis [1,2]. In addition, acute kidney injury after CABG can lead to the progression of CKD and the need for lifelong hemodialysis [2].

An *in situ* internal thoracic artery (ITA) graft is the most commonly used conduit during CABG due to its excellent long-term patency and fewer complications [3,4], and grafting of the left ITA to the left anterior descending artery is the first choice for CABG. However, there are concerns of a possible steal in patients with a functioning ITA graft and an ipsilateral upper-extremity VA creation [5]. Although some studies reported a hemodynamic coronary steal and consequent major adverse cardiac events (MACE) during hemodialysis in these patients [5–10], no reports definitely document the effects of the location (forearm *vs*. upper arm) and type (arteriovenous fistula [AVF] *vs*. prosthetic arteriovenous grafting [AVG]) of the VA on long-term clinical outcomes. The aims of the present study were to evaluate whether VA creation in the upper-extremity increases the risks of MACE and late death from any cause in patients with CABG with an *in situ* ITA graft, and to determine the clinical variables associated with MACE and late death in these patients.

## Materials and methods

### Study design and population

In this single-center, retrospective, observational cohort study, we analyzed data extracted from patient medical records; the study protocol was approved by our hospital’s institutional review board, and the approval included a waiver of informed consent. A total of 150 consecutive patients who underwent both isolated CABG and upper-extremity VA creation for hemodialysis, regardless of the time interval between these two procedures, between January 2001 and December 2014, were eligible. They comprised 3.4% of all patients undergoing VA creation at our hospital during the same period. Among them, 39 patients (26.0%) were excluded; 6 patients did not receive a left ITA graft during CABG, and 33 patients received hemodialysis *via* upper-extremity VA before CABG. In six patients in whom a left ITA graft was not used, five received a saphenous vein graft and one received a right ITA graft. In our analysis, there were no cases of CABG with *in situ* bilateral ITAs or right ITA by crossing the midline. The remaining 111 patients (74.0%) with CABG with an *in situ* left ITA graft who received the first upper-extremity VA creation followed by hemodialysis via a functioning VA were included, 93 (83.8%, ipsilateral group) of whom received left VA creation ipsilateral to a functioning left ITA graft and 18 (16.2%, contralateral group) who received right VA creation opposite to a functioning left ITA graft. Of the 18 patients receiving right VA creation, 3 were left-handed and 15 underwent left radial artery harvesting during previous CABG.

### VA creation

In this study, the preferred option for VA creation is an AVF, followed by an AVG on the most distal site of the nondominant arm that fulfilled the criteria for vessel suitability on either physical examination alone or duplex ultrasound on the basis of the guidelines, regardless of CABG [11]. All VA creation procedures were performed under local anesthesia, and VAs were categorized as AVF or AVG according to the type of VA. An AVF was created with a side-to-end anastomosis by using a 7/0 polypropylene continuous suture on the wrist or antecubital area; an AVG was created with 4–6-mm tapering polytetrafluoroethylene and inserted in a straight or U shape on the forearm or upper arm. There was no mortality or morbidity associated with VA creation. A functional VA was defined as allowing at least three adequate hemodialysis sessions.

### Study outcomes and follow-up

The ipsilateral and contralateral groups were retrospectively compared with regard to the following study outcomes: the primary outcome was defined as the occurrence of MACE including cardiac death, myocardial infarction, hospital admission for angina or heart failure, and repeat coronary revascularization; the secondary outcome was the composite of MACE or late death from any cause. Cardiac death was defined as any death related to cardiac events, including sudden death. Myocardial infarction was defined as any creatine kinase–myocardial band or cardiac troponin I increase above the upper limit of the normal range with either chest pain, symptoms consistent with ischemia, or electrocardiographic evidence of ischemia (i.e., ST-segment depression or elevation, or >1 mm elevation in two or more contiguous leads). Heart failure was defined as signs or symptoms of heart failure based on the Framingham score (two major or one major and two minor criteria) and evidence of systolic dysfunction (left ventricular ejection fraction ≤40%) on Doppler echocardiography (systolic heart failure), or as signs or symptoms of heart failure and evidence of diastolic dysfunction (passive transmitral left ventricular inflow velocity to tissue Doppler imaging velocity of the medial mitral annulus during passive filling [E/e’] ratio >15) (diastolic heart failure) [12–14]. Follow-up data were obtained from hospital charts or follow-up physicians, and by means of direct telephone interviews with the patients or their families. Only the first event of each outcome was included in the analysis. Follow-up ceased with the occurrence of MACE or late death from any cause or termination of VA.

Demographics, risk factors of interest, and other data were recorded for each patient. Prior cerebrovascular accidents (CVAs) were defined as a history of focal cerebral ischemia or hemorrhage; however, there was no hemorrhagic CVA in our analysis. Prior peripheral arterial occlusive disease (PAOD) was defined as radiological/surgical interventions for PAOD or an ankle brachial index of ≤0.9 as measured with Doppler ultrasound [15]. All data were collected, for all consecutive patients, in an Excel database (Microsoft Corp., Redmond, WA, USA), and the study outcomes and associated clinical variables were retrospectively analyzed.

### Statistical analysis

Categorical variables are reported as frequencies or percentages, and continuous variables as means and standard deviations. Categorical variables were compared for differences between the two groups by using chi-square test or Fisher’s exact test where appropriate, and continuous variables were compared for differences by using the Mann–Whitney *U*-test. Univariate and multivariate analyses of the association of clinical variables with the study outcomes were conducted with Cox proportional hazards modeling, by using the event of interest and period from study enrollment to the date of the event or last follow-up as the outcome. Univariate Cox proportional hazard regression models were fitted to calculate hazard ratios (HRs) with 95% confidence intervals (CIs) to estimate the association of clinical variables with the occurrence of primary or secondary outcome. Variables with a *P*-value of <0.3 on univariate analysis were included in multivariate Cox proportional hazard regression models. Long-term event-free rates were estimated with Kaplan–Meier analysis and were compared with that estimated with the log-rank test. *P*-values were two-tailed; *P*<0.05 was considered statistically significant. Statistical analyses were performed with SPSS version 21.0 (SSPS Inc., Chicago, IL, USA).

## Results and discussion

A total of 111 patients with CABG with an *in situ* left ITA graft who received upper-extremity VA creation were included in this study, and were stratified into the ipsilateral (93 patients, 83.8%; left VA creation) or contralateral (18 patients, 16.2%; right VA creation) groups based on the side of VA creation. The demographic data, risk factors, and clinical characteristics are summarized in **Table 1**. There were no significant differences in patient characteristics, with the exception of the VA location, between the ipsilateral and contralateral groups. Whereas 44.1% of the ipsilateral group received forearm VA, 72.2% of the contralateral group received forearm VA (*P* = 0.03). The types of VA (AVF *vs*. AVG) were similar between the two groups. The mean time interval from CABG to VA creation was 33.0±41.9 months, and no significant difference was noted between the two groups (32.1±43.1 *vs*. 37.8±37.8 months, *P* = 0.59). The mean follow-up period was similar between the two groups (37.7±27.5 *vs*. 39.1±35.8 months, *P* = 0.86).

**Table 1:** Baseline characteristics of the study population stratified by the side of VA creation.

	Ipsilateral group	Contralateral group	*P*-value
	(left-sided VA)	(right-sided VA)	
Number of patients	93	18	
Age, years	65.3±9.2	63.9±9.7	0.566
Male sex	62 (66.7)	14 (77.8)	0.353
BMI, kg/m^2^	24.0±3.1	24.3±3.8	0.711
Hypertension	83 (89.2)	14 (77.8)	0.238
Diabetes mellitus	78 (83.9)	15 (83.3)	0.999
Smoking	33 (35.5)	7 (38.9)	0.783
Dyslipidemia	12 (12.9)	4 (22.2)	0.290
MI	44 (47.3)	7 (38.9)	0.512
HF	16 (17.2)	5 (27.8)	0.327
CVA^a^	20 (21.5)	1 (5.6)	0.187
PAOD^a^	10 (10.8)	0 (0.0)	0.362
Time interval, months^b^	32.1±±43.1	37.8±37.8	0.594
Location of VA			0.029
Forearm	41 (44.1)	13 (72.2)	
Upper arm	52 (55.9)	5 (27.8)	
Type of VA			0.366
AVF	62 (66.7)	10 (55.6)	
AVG	31 (33.3)	8 (44.4)	
Follow-up period, months	37.7±27.5	39.1±35.8	0.858

AVF, arteriovenous fistula; AVG, arteriovenous graft; BMI, body mass index; CVA, cerebrovascular accident; HF, heart failure; MI, myocardial infarction; PAOD, peripheral arterial occlusive disease; VA, vascular access.

^a^History of CVA or PAOD.

^b^Time interval from coronary artery bypass grafting to VA creation.

**Fig 1:**
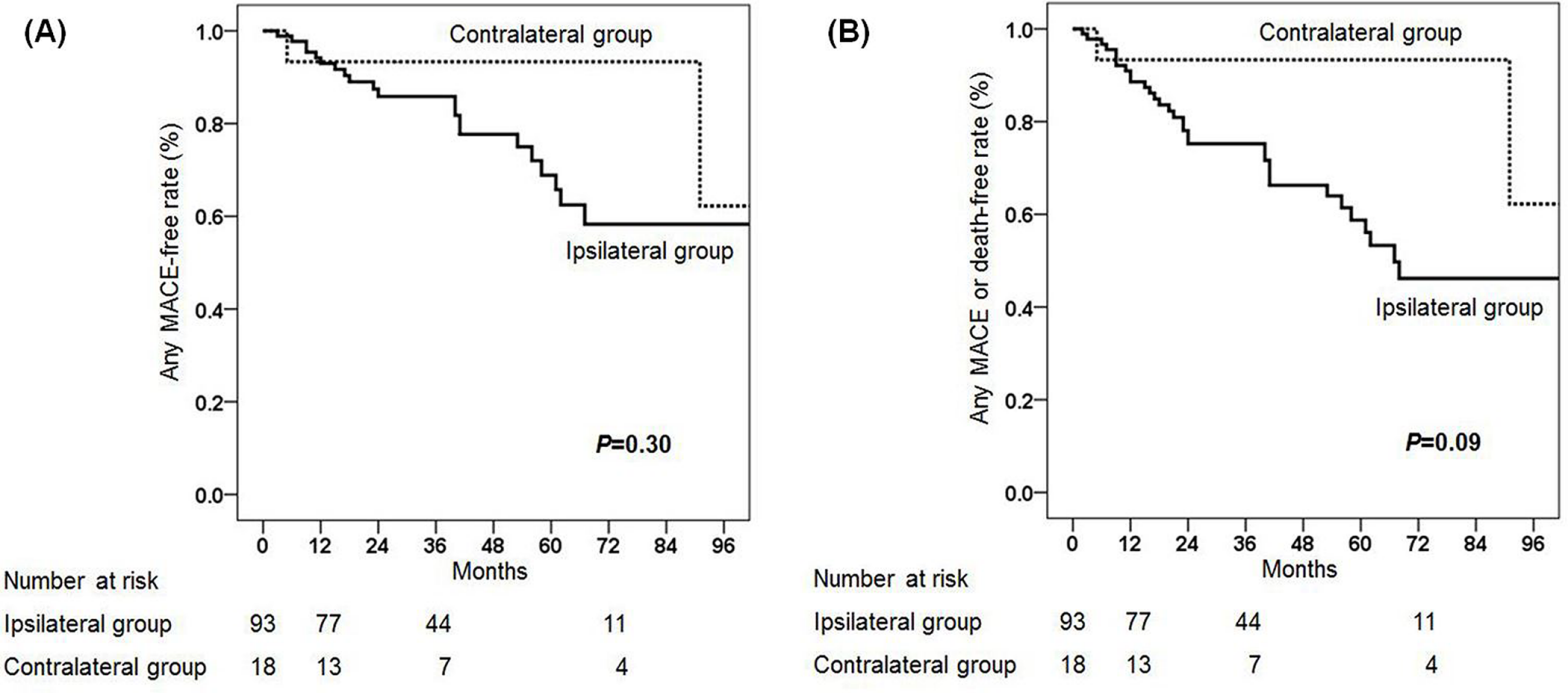
Kaplan-Meier survival analysis. Kaplan-Meier survival curve for **(A)** MACE-free and **(B)** the composite of MACE or late death-free rates in patients with ipsilateral and contralateral VA. MACE = major adverse cardiovascular events; VA = vascular access.

The primary outcome occurred in 24 patients (25.8%) in the group with ipsilateral and in 2 patients (11.1%) in the group with contralateral ITA grafts and VA, during a mean follow-up duration of 37.9±28.8 months. In the ipsilateral group, the primary outcome included cardiac death in 1 patient (1.1%), myocardial infarction in 13 patients (14.0%), hospital admission for angina or heart failure in 9 patients (9.7%), and repeat coronary revascularization in 1 patient (1.1%). In the contralateral group, one cardiac death (5.6%) and one hospital admission for angina or heart failure (5.6%) occurred during the same period. The secondary outcome occurred in 34 patients (36.6%) in the ipsilateral group and 3 patients (16.7%) in the contralateral group. The MACE-free (*P* = 0.30) and composite of MACE or late death-free (*P* = 0.09) rates were similar between the ipsilateral and contralateral groups (**Fig 1**). In our analysis, there was no VA-related hand ischemia in both groups during the follow-up period.

Multivariate Cox proportional hazard regression analysis, after adjusting for the confounding variables, indicated that prior CVA and the type of VA creation (an AVG) led to 3.30-fold (95% CI 1.37–7.97; *P* = 0.01) and 3.44-fold (95% CI 1.34–8.82; *P* = 0.01; **Table 2**) increased risk for the subsequent occurrence of the primary outcome, respectively, and prior PAOD was nonsignificantly associated with the primary outcome (HR 3.35; 95% CI 0.87–12.88; *P* = 0.08). Prior PAOD (HR 4.22; 95% CI 1.62–10.98; *P*<0.01) and the type of VA creation (an AVG) (HR 3.06; 95% CI, 1.42–6.61; *P*<0.01) emerged as statistically significant predictors of the subsequent occurrence of the secondary outcome, and prior CVA was nonsignificantly associated with the secondary outcome (HR 2.04; 95% CI 0.95–4.40; *P* = 0.07) (**Table 3**). The side and location of the VA were not associated with the occurrence of the primary or secondary outcomes.

**Table 2:** Clinical variables associated with the primary outcome.

	Univariate analysis		Multivariate analysis	
	HR (95% CI)	*P*-value	HR (95% CI)	*P*-value
Age, years	1.03 (0.98–1.07)	0.29	0.99 (0.94–1.05)	0.77
Male sex	1.58 (0.64–3.87)	0.32	NA	NA
BMI, kg/m^2^	0.95 (0.83–1.08)	0.43	NA	NA
Hypertension	0.74 (0.25–2.16)	0.58	NA	NA
Diabetes mellitus	0.75 (0.26–2.23)	0.61	NA	NA
Smoking	1.41 (0.63–3.19)	0.40	NA	NA
Dyslipidemia	0.45 (0.13–1.63)	0.23	1.01 (0.26–3.88)	0.99
MI	0.86 (0.38–1.94)	0.72	NA	NA
HF	1.02 (0.38–2.75)	0.97	NA	NA
CVA^a^	3.02 (1.33–6.85)	0.01	3.30 (1.37–7.97)	0.01
PAOD^a^	3.31 (0.96–11.39)	0.06	3.35 (0.87–12.88)	0.08
Time interval^b^	1.01 (1.00–1.02)	0.06	1.01 (1.00–1.02)	0.12
Side of VA				
Contralateral	Reference			
Ipsilateral	2.17 (0.51–9.32)	0.29	2.21 (0.45–10.85)	0.33
Location of VA				
Forearm	Reference			
Upper arm	0.93 (0.42–2.06)	0.86	NA	NA
Type of VA				
AVF	Reference			
AVG	3.23 (1.39–7.54)	0.01	3.44 (1.34–8.82)	0.01

AVF, arteriovenous fistula; AVG, arteriovenous graft; BMI, body mass index; CI, confidence interval; CVA, cerebrovascular accident; HF, heart failure; HR, hazard ratio; MI, myocardial infarction; NA, not applicable; PAOD, peripheral arterial occlusive disease; VA, vascular access.

^a^History of CVA or PAOD.

^b^Time interval from coronary artery bypass grafting to VA creation.

**Table 3:** Clinical variables associated with the secondary outcome.

	Univariate analysis		Multivariate analysis	
	HR (95% CI)	*P*-value	HR (95% CI)	*P*-value
Age, years	1.05 (1.01–1.09)	0.01	1.02 (0.97–1.06)	0.46
Male sex	1.34 (0.65–2.75)	0.43	NA	NA
BMI, kg/m^2^	0.93 (0.83–1.04)	0.19	0.98 (0.87–1.11)	0.79
Hypertension	1.14 (0.40–3.25)	0.80	NA	NA
Diabetes mellitus	1.24 (0.44–3.55)	0.68	NA	NA
Smoking	1.00 (0.50–2.01)	0.99	NA	NA
Dyslipidemia	0.33 (0.10–1.14)	0.08	0.75 (0.19–2.89)	0.67
MI	0.82 (0.42–1.62)	0.57	NA	NA
HF	0.84 (0.35–2.02)	0.69	NA	NA
CVA^a^	1.92 (0.93–3.95)	0.08	2.04 (0.95–4.40)	0.07
PAOD^a^	5.65 (2.40–13.30)	<0.01	4.22 (1.62–10.98)	<0.01
Time interval^b^	1.01 (1.00–1.01)	0.10	1.00 (1.00–1.01)	0.45
Side of VA				
Contralateral	Reference			
Ipsilateral	3.25 (0.78–13.61)	0.11	2.52 (0.56–11.48)	0.23
Location of VA				
Forearm	Reference			
Upper arm	0.97 (0.50–1.87)	0.92	NA	NA
Type of VA				
AVF	Reference			
AVG	3.27 (1.64–6.56)	<0.01	3.06 (1.42–6.61)	<0.01

AVF, arteriovenous fistula; AVG, arteriovenous graft; BMI, body mass index; CI, confidence interval; CVA, cerebrovascular accident; HF, heart failure; HR, hazard ratio; MI, myocardial infarction; NA, not applicable; PAOD, peripheral arterial occlusive disease; VA, vascular access.

^a^History of CVA or PAOD.

^b^Time interval from coronary artery bypass grafting to VA creation.

The results of our current study indicate that there were no significant differences in the incidence of primary and secondary outcomes between the group with ipsilateral and the group with contralateral ITA graft and VA. Whereas the side and location of the VA were not associated with an increased incidence of the primary or secondary outcome, the type of VA (an AVG) was significantly associated with increased risks of the primary or secondary outcome.

The ITA is most commonly used vascular conduit during CABG owing to its excellent late patency and fewer complications [3,4]. Moreover, the ITA could be used in patients with CKD because of its minimal atherosclerotic changes [16,17]. In addition to the occurrence of acute kidney injury after CABG, the increasing incidence of CKD and the extended life expectancy of patients undergoing CABG can lead to the progression of CKD and need for VA creation for lifelong hemodialysis [1,2]. The preferred option for VA creation in patients undergoing hemodialysis is a nondominant-upper-extremity AVF, followed by an AVG, because of their better outcomes and lower complication rates [11]. However, controversy exists about the application based on the guidelines and among previous studies in patients with CABG with an *in situ* left ITA graft when lifelong hemodialysis is necessary. Although there was no long-term follow-up, some studies reported that ipsilateral VA creation for hemodialysis can lead to a hemodynamic coronary steal from the ITA and consequent MACE during hemodialysis in patients with a functioning ITA graft, and concluded that hemodynamic changes from ipsilateral ITA flow caused by VA are potentially dangerous for these patients [5–10]. However, there were no guidelines or recommendations for the side (ipsilateral *vs*. contralateral), location (forearm *vs*. upper arm), or type (AVF *vs*. AVG) of VA creation in patients undergoing CABG referred for VA creation for hemodialysis.

There are two conflicting reports of long-term clinical outcomes in patients with both functioning VA for hemodialysis and CABG with a functioning ITA graft. Feldman et al. reported that upper-extremity VA for hemodialysis ipsilateral to an *in situ* ITA graft was associated with an increased risk of MACE during long-term follow-up [8]. On the other hand, Takami et al. reported that use of an ITA graft ipsilateral to the VA did not increase the operative mortality or the risk of late death and MACE after CABG in patients undergoing hemodialysis [5]. However, the patient populations were different between these two reports; one included 47 of 57 (82.4%) patients undergoing CABG before VA creation, and the other did not include patients undergoing CABG before VA creation. In our analysis, the study population comprised only patients with CABG before the construction of VA, and we focused on the planning of VA creation in patients undergoing CABG with a functioning ITA graft, based on the long-term clinical outcomes according to the side, location, and type of upper-extremity VA for hemodialysis.

The existence of a coronary steal is well documented in patients with proximal stenosis of the subclavian artery because both the functioning ITA graft and ipsilateral VA are supplied by the subclavian artery [18,19]. Several studies reported that ipsilateral VA and CABG with an ITA graft in patients with proximal subclavian artery stenosis induced a hemodynamic coronary steal and consequent cardiac ischemic symptoms during hemodialysis, and this steal syndrome was resolved after successful angioplasty of the stenotic subclavian artery [20,21]. They concluded that subclavian artery stenosis could play an important role in patients with CABG with an ITA graft and ipsilateral VA who had symptoms of a coronary steal syndrome. Therefore, the patency and degree of stenosis of the ipsilateral subclavian artery stenosis should be evaluated before the creation of an ipsilateral upper-extremity VA for hemodialysis in patients with CABG with an ITA graft. In our analysis, coronary artery angiography and/or coronary computed tomography angiography were performed before and after CABG, and there was no evidence of subclavian artery stenosis ipsilateral to the creation of VA in our study population.

The high morbidity and mortality in patients with CKD and CABG may result from more diffuse CAD with poor runoff [5]. However, Wong et al. reported that patients undergoing hemodialysis who had CABG did not have a greater coronary artery atherosclerosis burden than matched controls [22]. Therefore, the high morbidity and mortality in patients undergoing hemodialysis may be attributed to their extracardiac multiple comorbid disorders, including ventricular hypertrophy, CVA and PAOD, anemia, infection, electrolyte disturbances, cachexia, and increase in oxidative stress [23]. In our study, prior CVA and PAOD were significantly or nonsignificantly associated with increased risks for the subsequent occurrence of MACE and the composite of MACE or late death.

It is well known that AVFs are the preferred vascular access for chronic hemodialysis because of better outcomes, a lower complication rate once matured, and reduced costs compared with AVGs [11]. Furthermore, according to various operative techniques, VA could be associated with a potential risk of a coronary artery steal. It was postulated that high volume flow through the VA was associated with a coronary steal in patients with hemodialysis [24]. An AVF may be more likely to cause a coronary steal than an AVG because of an increased mean volume flow with time [25]. However, shortly after VA creation, especially in patients who already had CABG with an ITA graft, an ipsilateral AVG has greater hemodynamic effects on the cardiac and systemic circulation than an AVF with gradual increase of volume flow during the maturation process, which are more likely to cause adverse effects in these patients. In our study, although the side and location were not risk factors of the primary or secondary outcome, the type of VA (an AVG) was significantly associated with the increased incidence of the primary or secondary outcome.

This study has some important limitations of note. Because of its retrospective nonrandomized design with a small cohort of patients, this study was subject to considerable selection and information biases. There was no adjustment for sample size and baseline differences between the two groups, and only a few patients were included in the contralateral group because our policy is to create VA on the nondominant arm, regardless of CABG. Moreover, the hemodynamic values of the VA and *in situ* ITA graft were not measured, and we could not determine the hemodynamic effects of ipsilateral VA on the ITA graft, and the subsequent occurrence of MACE or late death in our analysis. Our findings were obtained at a single center, resulting in a small sample size and limiting the general relevance of our results. Our results may be affected by a small sample size. Future prospective trials with larger cohorts should lead to a better understanding of the impact of VA on the clinical outcomes in patients with CABG with an ITA graft.

In conclusion, our study showed no definite evidence that ipsilateral VA creation affects the subsequent occurrence of MACE or late death from any cause. However, it should be recognized that multiple atherosclerotic comorbid disorders, such as CVA and PAOD, are significant predictors of the subsequent occurrence of MACE or late death. Although the side and location of VA are not associated with MACE or late death, the type of VA (an AVG) is an important risk factor for the occurrence of MACE or late death in patients with CABG with an *in situ* ITA graft. Based on our analysis, in patients with CABG with an *in situ* ITA graft requiring upper-extremity VA creation for lifelong hemodialysis, we recommend that an AVF should be considered first rather than an AVG regardless of the side and location of VA, and in cases with no suitable veins for an AVF, a smaller tapering prosthetic graft should be used to minimize volume flow through the VA. Our findings may serve as novel data that could aid in the optimal operative strategy of upper-extremity VA creation for hemodialysis in patients with CABG with an *in situ* ITA graft.

## Supporting information

S1 Data Data of 111 patients with CABG with an *in situ* left ITA graft who received the first upper-extremity VA creation followed by hemodialysis via a functioning VA. (XLSX)
